# Consensus guidelines on the construct validity of rodent models of restless legs syndrome

**DOI:** 10.1242/dmm.049615

**Published:** 2022-08-10

**Authors:** Aaro V. Salminen, Stefan Clemens, Diego García-Borreguero, Imad Ghorayeb, Yuqing Li, Mauro Manconi, William Ondo, David Rye, Jerome M. Siegel, Alessandro Silvani, John W. Winkelman, Richard P. Allen, Sergi Ferré, the International Restless Legs Syndrome Study Group (IRLSSG)

**Affiliations:** 1 Institute of Neurogenomics, Helmholtz Zentrum München GmbH - German Research Center for Environmental Health, 85764 Neuherberg, Germany; 2 Institute of Human Genetics, Klinikum rechts der Isar, Technische Universität München, 81675 Munich, Germany; 3 Department of Physiology, Brody School of Medicine, East Carolina University, Greenville, NC 27834, USA; 4 Sleep Research Institute, 28036 Madrid, Spain; 5 Département de Neurophysiologie Clinique, Pôle Neurosciences Cliniques, CHU de Bordeaux, 33076 Bordeaux, France; 6 Université de Bordeaux, Institut de Neurosciences Cognitives et Intégratives d'Aquitaine, UMR 5287, 33076 Bordeaux, France; 7 CNRS, Institut de Neurosciences Cognitives et Intégratives d'Aquitaine, UMR 5287, 33076 Bordeaux, France; 8 Department of Neurology, Norman Fixel Institute for Neurological Diseases, College of Medicine, University of Florida, Gainesville, FL 32610, USA; 9 Sleep Medicine Unit, Regional Hospital of Lugano, Neurocenter of Southern Switzerland, 6900 Lugano, Switzerland; 10 Faculty of Biomedical Sciences, Università della Svizzera Italiana, 6900 Lugano, Switzerland; 11 Department of Neurology, University Hospital Inselspital, 3010 Bern, Switzerland; 12 Houston Methodist Hospital Neurological Institute, Weill Cornell Medical School, Houston, TX 77070, USA; 13 Department of Neurology, Emory University School of Medicine, Atlanta, GA 30322, USA; 14 Neuropsychiatric Institute and Brain Research Institute, University of California, Los Angeles, CA 90095, USA; 15 Neurobiology Research, Veterans Administration Greater Los Angeles Healthcare System, North Hills, CA 91343, USA; 16 Department of Biomedical and Neuromotor Sciences Alma Mater Studiorum, Università di Bologna, 48121 Ravenna Campus, Ravenna, Italy; 17 Departments of Psychiatry and Neurology, Massachusetts General Hospital, Harvard Medical School, Boston, MA 02114, USA; 18 Department of Neurology, Johns Hopkins University, Baltimore, MD 21224, USA; 19 Integrative Neurobiology Section, National Institute on Drug Abuse, Intramural Research Program, National Institutes of Health, Baltimore, MD 21224, USA

**Keywords:** Restless legs syndrome, Rodent models, Construct validity, Guidelines

## Abstract

Our understanding of the causes and natural course of restless legs syndrome (RLS) is incomplete. The lack of objective diagnostic biomarkers remains a challenge for clinical research and for the development of valid animal models. As a task force of preclinical and clinical scientists, we have previously defined face validity parameters for rodent models of RLS. In this article, we establish new guidelines for the construct validity of RLS rodent models. To do so, we first determined and agreed on the risk, and triggering factors and pathophysiological mechanisms that influence RLS expressivity. We then selected 20 items considered to have sufficient support in the literature, which we grouped by sex and genetic factors, iron-related mechanisms, electrophysiological mechanisms, dopaminergic mechanisms, exposure to medications active in the central nervous system, and others. These factors and biological mechanisms were then translated into rodent bioequivalents deemed to be most appropriate for a rodent model of RLS. We also identified parameters by which to assess and quantify these bioequivalents. Investigating these factors, both individually and in combination, will help to identify their specific roles in the expression of rodent RLS-like phenotypes, which should provide significant translational implications for the diagnosis and treatment of RLS.

## Abstract (precis)

**Summary:** We summarize the consensus guidelines on the construct validity of rodent models of restless legs syndrome, which have been recently established by a task force of the International Restless Legs Syndrome Study Group.

## Introduction

Restless legs syndrome (RLS), also known as Willis-Ekbom disease, is a common sensorimotor disorder with a prominent circadian pattern. According to the RLS Epidemiology, Symptoms and Treatment (REST) study, about 5% of US and European adults reported experiencing RLS symptoms at least weekly (Allen et al., 2005). RLS is characterized by a rest-induced, movement responsive, mostly nocturnal, urge to move the legs or akathisia, which in RLS is typically associated with uncomfortable or unpleasant sensations in the legs (Allen et al., 2014). Sleep disruption is the primary factor producing most of the morbidity (Kushida et al., 2004), but RLS patients usually do not report sleepiness during daytime, despite their reduced total sleep time (Allen et al., 2010).

RLS diagnosis, therefore, relies upon subjective clinical features or symptoms (Box 1; Allen et al., 2014). Objective clinical features or signs, such as periodic limb movements (PLM) during sleep (PLMS), are only included as features supportive of diagnosis (Box 1; Allen et al., 2014). The fact that an RLS diagnosis mostly depends on subjective clinical features is a significant challenge for preclinical research. Addressing this issue is particularly challenging when developing animal models of RLS with *face validity*, that is, animal models that closely reproduce the clinical features of human RLS. The challenge of defining parameters for rodent models of RLS with face validity was recently addressed by a task force convened by the International Restless Legs Syndrome Study Group (IRLSSG) (Salminen et al., 2021).

**Box 1. International RLS Study Group (IRLSSG) consensus diagnostic criteria for RLS**

Essential diagnostic criteria:

- The urge to move the legs usually but not always accompanied by, or felt to be caused by, uncomfortable and unpleasant sensations in the legs.
- The urge to move the legs and any accompanying unpleasant sensations begin or worsen during periods of rest or inactivity, such as lying down or sitting.
- The urge to move the legs and any accompanying unpleasant sensations are partially or totally relieved by movement, such as walking or stretching.
- The urge to move the legs and any accompanying unpleasant sensations during rest or inactivity only occur or are worse in the evening or night than during the day.
- The occurrence of the above features is not solely accounted for as symptoms primary to another medical or behavioral condition (e.g. myalgia, venous stasis, leg edema, arthritis, leg cramps, positional discomfort, habitual foot tapping).

Clinical features supporting the diagnosis of RLS:

- Periodic limb movements (PLM); presence of PLM during sleep or wakefulness (PLMS or PLMW, respectively) at a rate or intensity that is greater than expected for age or medical and medication status.
- Dopaminergic treatment response; reduction in symptoms at least initially upon treatment with low doses of dopaminergic agonists.
- Family history of RLS among first-degree relatives.
- Lack of profound daytime sleepiness.

In addition to face validity, a valid translational rodent model should also have construct validity. Construct validity considers how well the mechanisms used to induce the clinical features of a (neuropsychiatric) disorder in an animal model reflect its currently understood pathophysiology (Salminen et al., 2021). Doing so requires an in-depth understanding of the biological basis of a human disorder. However, an additional challenge for RLS preclinical research is the lack of consensus about its main risk and triggering factors, and pathophysiological mechanisms. These challenges hinder reaching an agreement about the *construct validity* of RLS animal models.

The IRLSSG task force that established guidelines for rodent models of RLS with face validity reconvened to establish guidelines for construct validity. The starting point was to determine the most accepted risk and triggering factors, and the pathophysiological mechanisms of RLS. Selected factors and biological mechanisms were then evaluated for their possible translation in rodent models of RLS. Recommendations were then presented to assess and quantify these parameters and to identify potential approaches to induce or reproduce them in rodents.

## Methods used to determine RLS models with construct validity

Following the compilation of the IRLSSG consensus guidelines on rodent models of RLS (Salminen et al., 2021), the IRLSSG Executive Committee approved a motion in October 2020 that the previously appointed animal models task force continue its work and assess the construct validity of potential RLS models. Twelve of the original 14 members undertook this work. As previously reported, a modified Delphi method (Salminen et al., 2021) was used to reach a consensus and to ensure anonymity. Following an initial telephone conference during which all participants approved the aims and the general methodology of the task force, three phases of guideline development were then conducted through e-mail correspondence, with the assistance of a facilitator.

### Phase one

This phase involved the development of a list of potential risk or triggering factors and pathophysiological mechanisms of RLS in humans, based on the existing literature. In the first round, each task force member submitted a list of objective clinical findings, including but not restricted to genetic, analytical, imaging and pathological findings, that they posited to be associated with RLS. To facilitate the identification of missing items, the list was categorized before undergoing a second round of suggestions to give each task force member the opportunity to add any potentially overlooked items. To promote discussion, members were permitted to write counterpoints to any items that they believed should be removed and rebuttals to any of the counterpoints that had been made. Each task force member then voted anonymously for the inclusion or exclusion of each item on the list. Items receiving ≤50% of votes were excluded. The remaining items were categorized into one of three categories: risk factors, trigger factors and pathophysiological mechanisms, and ranked according to the percentage of positive votes received.

### Phase two

In the second phase, the leading committee (A.S., S.C., S.F.) translated, where possible, each item into factors relevant to rodent models. These translations were circulated among the task force for discussion.

### Phase three

Using the list of translated items, the task force developed actionable guidelines. In the first round of this phase, the leading committee proposed methods to measure each of the previously identified factors in a rodent model, as well as methods that could be used to induce or reproduce each feature in rodents, as specifically as possible. Modifications were made following a review by the task force, and a final version was unanimously accepted. After approval of the written report by all task force members, the recommendations were forwarded to the IRLSSG Executive Committee for review and endorsement.

## RLS risk factors, triggering factors, and pathophysiological mechanisms

The task force suggested a total of 51 options for the final list of RLS risk factors, triggering factors and pathophysiological mechanisms; of these, 31 were excluded by consensus, resulting in a final list of 20 items, which the task force considered to have sufficient support in the literature. These items were classified into six groups, which we discuss below.

### Genetic and sex-related factors

**Fig. 1.**
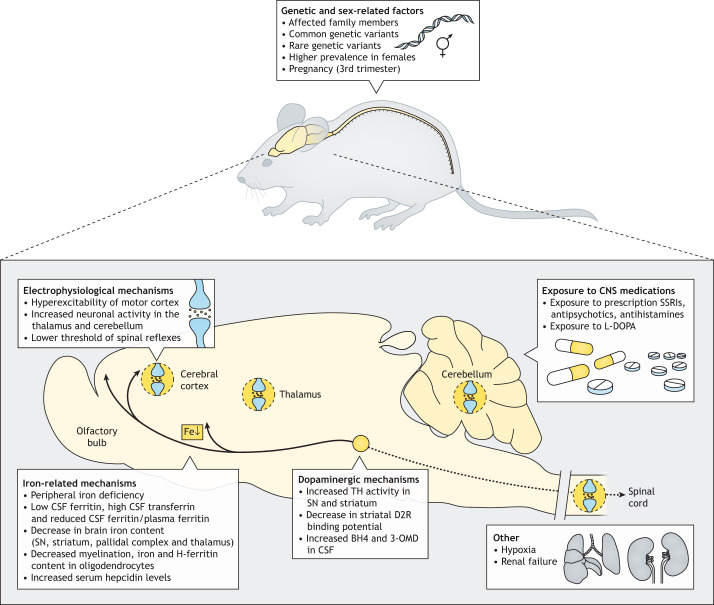
**RLS risk and triggering factors and pathophysiological mechanisms.** Schematics of selected risk factors, triggering factors and pathophysiological mechanisms in RLS, which should be implemented or reproduced in rodents to provide animal models of RLS with construct validity. The schematic at the bottom depicts a rodent brain, with anterior to the left and dorsal uppermost, and the caudal section of the spinal cord. It shows factors, such as hyperexcitability in the frontal cortex, thalamus, cerebellum and spinal cord, involvement of the ascending dopaminergic system and possible additional involvement of the descending dopaminergic system, hypoxia (lungs) and renal insufficiency (kidneys). Abbreviations: BH4, tetrahydrobiopterin; CNS, central nervous system; CSF, cerebrospinal fluid; D2R, dopamine D_2_ receptor; Fe, iron; 3-OMD, 3-ortho-methyl-DOPA; SN, substantia nigra; SSRIs, selective serotonin reuptake inhibitors; TH, tyrosine hydroxylase.

Genetic and sex-related factors include affected family members, common genetic variants, rare genetic variants, higher prevalence of RLS in females, and pregnancy (Fig. 1A).

RLS is a complex genetic disorder; its heritability is determined by a combination of genetic risk variants and non-genetic environmental factors (Schormair and Winkelmann, 2011). Family and twin studies have estimated that the heritability of RLS is 50–60% (Schormair and Winkelmann, 2011). The genetic risk factors of RLS can be divided into common and rare, according to the frequency of the respective risk allele (Schormair and Winkelmann, 2011). Common genetic variants associated with human disease can be identified in genome-wide association studies (GWAS) and can influence disease progression with low penetrance by affecting the expression patterns of target genes, for example, when localized in regulatory elements. Two recent GWAS meta-analyses confirmed a total of 23 RLS risk variants in 22 genomic risk loci, including the first-reported RLS risk loci near *MEIS1*, *BTBD9* and *PTPRD* (Schormair et al., 2017; Didriksen et al., 2020). However, caution should be applied when interpreting the effects of common variants, as the affected gene might not be the gene that is nearest to the associated genetic locus (Mumbach et al., 2017). Rare variants, detected by targeted sequencing or by whole-exome or whole-genome sequencing, are often located in the coding regions of the genome (Schormair and Winkelmann, 2011). These variants may have a direct effect on the amino acid composition and function of the encoded protein.

RLS has a higher prevalence among women than men (Manconi et al., 2012). Hormonal differences between men and women are unlikely to account for this difference (Ghorayeb et al., 2008) but might, instead, be partly due to pregnancy (Pantaleo et al., 2010), as a high prevalence of RLS occurs during the third trimester of pregnancy (Darvishi et al., 2020). Furthermore, even though RLS regularly resolves upon delivery, women who develop RLS symptoms during pregnancy are, compared to nulliparous women, up to four times more susceptible to developing RLS later in life (Cesnik et al., 2010; Earley et al., 2014). Therefore, pregnancy is a triggering factor for RLS, whereas previous pregnancies are a risk factor for the increased susceptibility to developing RLS again in the future.

### Iron-related mechanisms

Iron-related factors for RLS include: peripheral iron deficiency (PID); low levels of cerebrospinal fluid (CSF) ferritin, high levels of CSF transferrin and a reduced CSF ferritin-to-plasma ferritin ratio; decrease in brain iron content, particularly in the substantia nigra (SN), striatum, pallidal complex and thalamus; decrease in iron content and decrease in H-ferritin, predominantly in oligodendrocytes; decreased myelination; and increased levels of serum hepcidin (Fig. 1B).

Iron-related findings for RLS can be grouped into CSF/plasma, post-mortem and imaging findings, which provide significant evidence that brain iron deficiency (BID) is often involved in the pathophysiology of RLS (Earley et al., 2014; Ferré et al., 2019). PID, in contrast, constitutes a risk or triggering factor, enabling the development of BID in a vulnerable individual. The prevalence of RLS in patients with anemia secondary to iron deficiency is as high as 30% (Allen et al., 2013a), i.e. six times higher than the prevalence of RLS in the general population (Allen et al., 2005).

Nevertheless, many RLS patients do not show systemic iron deficiency but rather appear to have a specific iron deficiency in the brain, substantiated by relatively lower CSF ferritin, higher CSF transferrin and reduced CSF ferritin/plasma ferritin levels compared to those of controls (Earley et al., 2000; Mizuno et al., 2005), as well as decreased iron content of the SN, striatum, pallidal complex and thalamus (Allen et al., 2001; Godau et al., 2007, 2008; Rizzo et al., 2013; Li et al., 2016). Although less frequently, negative findings have also been reported (Knake et al., 2009; Margariti et al., 2012).

In post-mortem tissue, this specific iron deficiency in the brain appears as decreased levels of iron and H-ferritin, predominantly in oligodendrocytes (Connor et al., 2003, 2011a), the brain cells with the highest iron and ferritin content, and which synthesize myelin (Todorich et al., 2009). Decreased myelination in RLS might, thus, arise from an iron-dependent impairment of oligodendrocyte function (Connor et al., 2011b). Hepcidin is a central regulatory molecule of systemic iron homeostasis, which is secreted by the liver and decreases systemic iron levels (Hentze et al., 2010). Increased serum hepcidin levels without a concomitant decrease in plasma iron concentrations have been reported in RLS patients (Dauvilliers et al., 2018; Chenini et al., 2020) and imply the existence of a not-yet-understood dysregulation of systemic iron metabolism in RLS. However, one recent report does not differentiate between plasma hepcidin levels in RLS patients and in controls from a large population of blood donors (Dowsett et al., 2021).

### Electrophysiological mechanisms

Electrophysiological RLS-related factors include: hyperexcitability of the motor cortex, increased neuronal activity in the thalamus and cerebellum, and lower threshold of spinal reflexes (Fig. 1C).

The most-important evidence for hyperexcitability of the motor cortex in RLS patients stems from transcranial magnetic stimulation (TMS) studies, which indicate increased excitability in the motor cortex with reduced intracortical inhibition (Tergau et al., 1999; Quatrale et al., 2003; Scalise et al., 2004; Lanza et al., 2015; Magalhães et al., 2019). Most of these studies conclude that the pyramidal tract is intact in RLS patients, and that altered cortical excitability depends on cortical and subcortical mechanisms. Importantly, a TMS study reported a predominant increase in excitability within the part of the primary motor cortex representing the leg (Salas et al., 2018).

Functional magnetic resonance imaging (fMRI) has repeatedly shown significantly increased activation of the thalamus and cerebellum in RLS patients (Bucher et al., 1997; Margariti et al., 2012; Zhuo et al., 2017). Neuronal overactivity in the thalamus of RLS patients is also supported by results obtained by using proton magnetic resonance spectroscopy (MRS), which showed an increased Glx signal, i.e. the combined signal of glutamine and glutamate, in RLS patients compared to that in controls (Allen et al., 2013b). The increased excitability of the motor cortex, and increased neuronal activity of the thalamus and cerebellum observed in studies using TMS, fMRI and MRS were independent of the presence of RLS symptoms. Decreased concentrations of N-acetylaspartate without neuronal loss, indicative of neuronal dysfunction, have been specifically detected in the medial thalamus of RLS patients (Rizzo et al., 2012). This thalamic area includes the midline–intralaminar nuclei, which project to the cortex and striatum (Groenewegen and Berendse, 1994), and which are directly connected to the dentate nucleus of the cerebellum (Bostan and Strick, 2018).

RLS is nearly always associated with PLM, which, together with spinal reflexes, are in large part mediated by neural circuits in the spinal cord (Telles et al., 2011). A functional role for the spinal circuits in the emergence of RLS symptoms is supported by data that have identified altered spinal cord flexor withdrawal reflexes in RLS, where spinal reflex excitability is often increased in the evening during both wakefulness and sleep (Dafkin et al., 2017, 2018; Bara-Jimenez et al., 2000; Aksu and Bara-Jimenez, 2002; Abdulhadi et al., 2021). Additional evidence comes from findings that PLM can occur independently of descending control after spinal cord injury (Telles et al., 2011) and can improve after treatment with dopaminergic agonists (Salminen et al., 2013).

### Dopaminergic mechanisms

Dopaminergic RLS-related factors include: increased levels of tetrahydrobiopterin (BH4) and 3-ortho-methyl-DOPA (3-OMD) in the CSF, increased activity of tyrosine hydroxylase (TH) in the SN and striatum, and decreased binding potential of striatal dopamine D_2_ receptor (D2R) (Fig. 1D).

CSF, post-mortem, and imaging findings indicate that increased dopamine synthesis and release occurs in the brains of RLS patients. Increased BH4 and 3-OMD levels in the CSF of RLS patients has been reported in several studies (Earley et al., 2001, 2006; Allen et al., 2009). Increased expression of active (phosphorylated) TH in the SN (pars compacta) and striata of RLS patients has been reported in a study where the same results were demonstrated in rats with diet-induced BID (Connor et al., 2009), strongly suggesting a connection between BID and the increase in dopamine synthesis and release. Positron emission tomography (PET) and single-photon emission computed tomography (SPECT) studies have assessed the status of D2R in the striata of patients with RLS, with most but not all studies showing a decrease in D2R ligand binding (reviewed in Earley et al., 2014). A more-recent combined PET/SPECT study showed evidence for a decrease in the D2R binding potential being mostly dependent on increased synaptic dopamine (Earley et al., 2013), which is compatible with increased dopamine synthesis and release. This apparent ‘cerebral hyperdopaminergic state’ associated with RLS seems counterintuitive, given the effective therapeutic response to low doses of dopaminergic agonists.

However, this apparent ‘dopaminergic paradox’ can be explained by their effect on presynaptic D_2_-like receptors (D2R, D3R or D4R subtypes) (Ferré et al., 2021). D_2_-like receptors that localize to dopaminergic cells (autoreceptors) exert a strong inhibitory control on their neuronal activity and mediate the decreased locomotor activity in rodents that is induced by low doses of non-selective D_2_-like receptors (Maj et al., 1997; Chernoloz et al., 2009; Li et al., 2010). Results obtained in rats with diet-induced BID also indicate a significant role for presynaptic D_2_-like receptors in RLS that localize to cortico-striatal glutamatergic terminals (Yepes et al., 2017). Finally, the spinal cord, which receives descending dopaminergic innervation, represents another plausible localization of D_2_-like receptors involved in the therapeutic effects of dopaminergic agonists for RLS (Thorpe et al., 2011).

### Exposure to CNS-activating medications

Exposure to prescribed selective serotonin reuptake inhibitors (SSRIs), antipsychotics or antihistamines as well as exposure to L-DOPA is linked to RLS (Fig. 1E).

RLS can develop owing to exposure to several CNS-active medications, such as SSRIs (Yang et al., 2005; Rottach et al., 2008), that increase the extracellular levels of serotonin, and can secondarily exert excitatory effects on the dopaminergic system (Howell and Cunningham, 2015). Antipsychotic treatment has also been associated with RLS (Patatanian and Claborn, 2018) but is more commonly found together with akathisia, i.e. the inability to remain still, but without the unpleasant sensations in the legs and the circadian component of RLS. This has been suggested to depend on a blockade of presynaptic dopamine receptors in the ventral striatum (Ferré et al., 2021). Treatment with antihistamines, particularly those that cross the blood-brain-barrier and bind to the H_1_-receptor subtype, can markedly worsen RLS symptoms (Mackie and Winkelman, 2015). This outcome may also be related to the ability of antihistamines to interact with the dopaminergic system (Oleson et al., 2012; Flik et al., 2015).

Levodopa (L-DOPA) and several D_2_-like receptor agonists are, initially, very effective therapeutic agents for RLS. They do not trigger RLS but lead to a worsening of symptoms over time, a phenomenon known as ‘augmentation’ (Allen and Earley, 1996; Allen et al., 2011).

### Other factors

Other factors linked to RLS include hypoxia and renal failure (Fig. 1F). Peripheral hypoxia, as measured in the legs during the symptomatic period, is more frequent in patients with RLS and correlates with symptom severity (Salminen et al., 2014). Markers of hypoxia are also overexpressed in the skeletal muscles of RLS patients (Wåhlin-Larsson et al., 2009). Association of RLS with systemic hypoxia is supported by its association with prolonged exposure to high altitudes (Gupta et al., 2017) and with its recently described moderate, but significant, increased association with chronic obstructive pulmonary disease (Thi Truong et al., 2021). In addition, the activation of hypoxic pathways has been demonstrated in the SN and brain microvasculature of RLS patients (Patton et al., 2011).

The prevalence of RLS among patients suffering from renal insufficiency may be ≤30% (Lin et al., 2019), significantly higher than in the general population. In some cases, RLS resolves after a successful kidney transplant (Capelli et al., 2019), supporting the causal relationship between the two conditions. Therefore, renal failure may be classified as a triggering factor for RLS.

## Translation of risk factors, triggering factors and pathophysiological mechanisms into rodent models

Next, the task force attempted to translate the selected items into rodent models and proposed guidelines on how to both measure and induce or reproduce these factors in rodent models.

### Genetics and sex-related factors

#### Affected family members

As noted in our previous guidelines (Salminen et al., 2021), rodents of an inbred colony are genetically identical to each other and share an identical environment. Therefore, observation and comparison of rodent families would not be as applicable as they are when using clinical cohorts. Instead, the focus of rodent studies should be on the specific genetic and environmental causes that influence the heritability of RLS.

#### Common genetic variants

Common genetic variants can often not be translated directly to rodents owing to the incomplete conservation of non-coding regions of the genome between species, but humans may share more genomic commonalities with other species than previously thought (Leypold and Speicher, 2021). RLS research should focus on the effect that a specific variant has on gene expression. When the expression of a target gene is downregulated by the risk allele of the variant, heterozygous or homozygous knockout (KO) models of the target gene may be considered. This approach has been used extensively in RLS animal models (DeAndrade et al., 2012; Drgonova et al., 2015; Salminen et al., 2017). Gene silencing methods, such as RNA interference, may also be considered in both *in vivo* and *in vitro* models. When an RLS risk variant is associated with the upregulation of a target gene, overexpression approaches should be used. If tissue-specific effects on gene expression are known to be associated with the variant, conditional KO systems specific to the organs or cell types of interest should be considered. The most common tool used to generate tissue-specific genetic modifications in rodents is the Cre-LoxP system (Tsien, 2016). This approach may also be used when a hypothesis concerning a disease-relevant tissue has been established. Conditional genetic modifications using the Cre-LoxP system have already been used in RLS animal models (Lyu et al., 2019a, 2020a). If the non-coding variant is located within a highly conserved regulatory region that is also identified in the rodent genome, the genetic manipulation of those regulatory elements may be considered.

#### Rare genetic variants

Rare genetic variants can, in some cases, be directly translated to rodent models. When sufficient inter-species conservation of the target region is present, an individual variant may be introduced into the rodent genome by using gene-editing technology, such as CRISPR-Cas9. When an exaggerated effect of the variant is warranted, a larger part of the genetic sequence or an entire domain of the affected protein could be altered. However, such approaches may introduce unwanted effects on protein folding and would need to be well validated. If modification of gene expression is preferred, similar approaches to those outlined above for common genetic variants can be used.

#### Higher prevalence in females

As recommended in our previous guidelines (Salminen et al., 2021), both sexes should be used in RLS animal experiments because of the many sex-related biological differences. That sex-related differences may be driven by previous pregnancies, as in humans, should also be considered.

#### Pregnancy (third trimester)

The biological differences between human and rodent pregnancies are significant, ranging from the typical number of offspring per pregnancy to the timing of delivery relative to the development of the offspring. Although technically possible, monitoring rodents during pregnancy can be challenging for practical and ethical reasons. The time of pregnancy onset should be determined by using a vaginal plug. If possible, the litter size should be recorded and controlled for by data analysis. Pregnant rodents can be observed during the second half of their pregnancy. However, this may not correspond biologically to the third trimester of human pregnancy and, therefore, the interpretation of such results is challenging. One study has investigated the motor behavior of pregnant rats as a potential model for RLS (Mariano et al., 2014).

### Iron-related mechanisms

#### Peripheral iron deficiency

PID may be indirectly assessed by measuring hemoglobin levels. However, the analysis of serum ferritin more accurately evaluates non-anemic PID (Quiroz et al., 2016; Lo Martire et al., 2018; Zhu et al., 2020). An iron-deficient diet can induce PID without concomitant BID in the adult rodent, which is – unlike weaning animals − very resistant to BID (see section below).

#### Low CSF ferritin, high CSF transferrin and reduced CSF ferritin-to-plasma ferritin levels

In human RLS patients, these measures are used as indirect markers of general BID and may be analyzed in rodents. However, rodent models allow for more-direct measurements of BID (see below). Currently, in the most-used animal model of BID, i.e. the diet-induced BID model, rodents are exposed to a strict iron-deficient diet during the post-weaning period, a time when they are specifically sensitive to developing BID (Earley et al., 2014). However, this approach is associated with PID, which could determine or modify the experimental results. Inbred strain recombination has provided mouse strains with a predominant BID versus PID upon an iron-deficient diet during the post-weaning period, such as females of the BXD S40 mouse strain (Jellen et al., 2009, 2012; Allen et al., 2020). The BXD S40 female mouse might, therefore, represent a better model for RLS with regards to BID, since it could provide clues about the mechanisms involved in the specific dysregulation of iron levels within the brain during RLS, as well as identify the loci that cause the BXD 40 strain to exhibit such dysregulation, thereby helping to identify orthologous loci influencing RLS in human patients. Mice with non-anemic BID and PID also have been obtained by using a less-severe but longer-lasting iron-deficient diet during the post-weaning period (Quiroz et al., 2016).

#### Decrease in brain iron content

Decreased iron levels, particularly in the SN, striatum, pallidal complex and thalamus, can be directly determined by measuring the iron content, or the density or expression of the transferrin receptor, which is specifically upregulated upon chronic cellular iron deficiency (Lok and Loh, 1998; Han et al., 2003; Gulyani et al., 2009). A strong inverse correlation between iron content and density or mRNA expression of the transferrin receptor protein has been demonstrated in the rat brain, indicating that these factors can be used as indirect measures of BID in rodents (Han et al., 2003). Iron content can be measured histochemically by Perls Prussian Blue staining or by mass spectrometry, and transferrin receptor density or expression can be measured by immunohistochemistry, western blotting, quantitative PCR (qPCR) or RNA *in situ* hybridization, e.g. RNAscope (Connor et al., 2011a; Quiroz et al., 2016; Han et al., 2003; Gulyani et al., 2009). The SN (pars reticulata), striatum, pallidal complex and thalamus are brain areas with the highest iron content, and appear to experience a more-profound decrease in iron content than other brain areas upon BID in RLS (Allen et al., 2001; Godau et al., 2007, 2008; Rizzo et al., 2013; Li et al., 2016). Therefore, a more-profound decrease in brain iron content in these areas should also be expected in the BID rodent. The specific role of iron deficiency within each distinct brain area in the pathophysiology of RLS symptomatology could be evaluated by local infusion of iron chelators, such as deferiprone. This approach has been recently used to study axonal iron transport between different areas of the mouse brain (Wang et al., 2019). Genome editing tools could also be used to induce a decrease of intracellular iron content in specific cells, such as neurons or glia, and in brain areas, by using Cre-LoxP-mediated recombination techniques, e.g. by conditionally knocking down the expression of transferrin receptors.

#### Decreased myelination, and reduced iron and H-ferritin content in oligodendrocytes

Iron content can be measured histochemically by Perls Prussian Blue staining, and markers of H-ferritin and oligodendrocytes by immunohistochemical techniques (Connor et al., 2011a; Wan et al., 2020). H-ferritin from isolated crude myelin fractions extracted from brain tissue can also be analyzed in western blotting (Connor et al., 2011b). Oligodendrocytes are the producers of myelin in the CNS and are the cells with the highest iron content in the CNS (Todorich et al., 2009). They also seem to experience a more-profound decrease in iron content than other cell types upon BID in RLS (see above). Their specific role in the pathophysiology of RLS symptomatology could be evaluated in transgenic rodents by using selective and conditional KO of H-ferritin in oligodendrocytes. Such a conditional KO has recently been published and shows severe oligodendrocyte dysfunction and hypomyelination, particularly when the selective genetic blockade of H-ferritin expression occurs during the post-weaning period (Wan et al., 2020).

#### Increased serum hepcidin levels

Serum hepcidin levels can be directly measured in rodents. Transgenic mice that overexpress hepcidin in their hepatocytes have been recently generated (Zhang et al., 2018). These animals show a significant increase in serum hepcidin levels and could be used to study a possible pathogenetic mechanism of increased hepcidin.

### Electrophysiological mechanisms

#### Hyperexcitability of the motor cortex

*In vivo* techniques that track neuronal activation in rodents include manganese-enhanced MRI, an approach that has already been used to study cortical hyperexcitability in a genetic rodent model of RLS (Lyu et al., 2020a); [^18^F]-fluorine-labeled 2-fluoro-2-deoxy-D-glucose PET imaging of metabolic activity (Cai et al., 2019); and the use of miniaturized fluorescence microscopes (miniscopes) or fiber photometry, both of which measure fluctuations of calcium ions (Ca^2+^) (Girven and Sparta, 2017; Shiromani et al., 2021). Optogenetic stimulation combined with *in vivo* microdialysis/voltammetry/fiber photometry can be used to determine an increased sensitivity of motor cortical pyramidal cells to the release of glutamate by their nerve terminals in the striatum or spinal cord (Yepes et al., 2017). *In vitro* techniques could be used to measure the neuronal properties at both the somatodendritic and nerve-terminal level. These techniques include electrophysiological analysis of *ex vivo* cortico-striatal slices, and measurement of intrinsic excitability and spontaneous firing activity, as well as electrically or optogenetically induced pre- and postsynaptic events (i.e. the probability of neurotransmitter release and excitatory postsynaptic currents) (Lyu et al., 2020a). The hyperexcitability of the motor cortex could be induced by using Cre-LoxP recombination techniques to promote the selectively targeted expression of ion channel subunits within the motor cortex that can alter neuronal excitability. This approach could generate, for example, a gain-of-function sodium channel in glutamatergic neurons or a loss-of-function potassium channel in GABAergic neurons. This strategy has been used in global knock-in mice – without a targeted expression to specific neuronal populations – to obtain genetic rodent models of epilepsy (Oyrer et al., 2018). As a more-translational method, experimental repetitive TMS (rTMS) was recently introduced in awake rats to promote the focal stimulation of the motor cortex (Cermak et al., 2020), and has been used clinically to modify cortical excitability (Lanza et al., 2018). Optogenetics and ultrasound techniques are alternative methods that could also be used *in vivo* to increase neuronal excitability of the motor cortex through repetitive stimulation or other stimulation techniques (Magno et al., 2019; Qiu et al., 2021).

#### Increased neuronal activity in the thalamus and cerebellum

The imaging and miniscope approaches discussed above are used to measure neuronal activation in the motor cortex but could also be used to measure neuronal activation in thalamic and cerebellar areas. The same *in vivo* techniques, such as PET imaging, miniscope, fiber photometry, optogenetics, *in vivo* microdialysis, as well as *in vitro* techniques, such as brain slice electrophysiology, could be applied to the midline and intralaminar thalamic nuclei and their glutamatergic outputs to the striatum and cortex (Groenewegen and Berendse, 1994), and their glutamatergic input from the cerebellar dentate nucleus (Bostan and Strick, 2018). Similarly, the same approaches suggested to induce motor cortex hyperexcitability – excluding rTMS, which cannot target deep and discrete brain areas – could also be used to induce increased neuronal activity in the thalamic and cerebellar nuclei.

#### Lower threshold of spinal reflexes

Thresholds of spinal cord reflexes can be readily assessed in rodents both *in vivo* and *in vitro*. *In vivo*, this can involve the assessment of thermal withdrawal latencies in awake animals by using a Hargreaves or a tail-flick assay (Brewer et al., 2014; Rodgers et al., 2019). Sensory stimulation can also be performed in anesthetized animals to identify the responses of specific neuronal populations (Douglass and Carstens, 1997). In addition to thermal withdrawal reflexes, behavioral assessments can test for changes in mechanoreceptor sensitivity using von Frey filaments (Christensen et al., 1996). Another approach would be to implant intrathecal minipumps to deliver a drug with a high spatial resolution to specific parts of the spinal cord (Dableh et al., 2009). Surgical interventions, such as spinal transection, can be performed to evaluate the role of the sensorimotor circuitry *in vivo* without the influence of a descending modulatory control (Kisucká et al., 2015). In addition, interventional approaches enable the study of the correlation of spinal with supraspinal mechanisms (Watanabe et al., 2015). Spinal cords can also be readily isolated *in vitro* and placed in a dish with artificial cerebrospinal fluid, where sensory input can be electrically stimulated and the elicited motor outputs recorded (Brewer et al., 2014; Machacek et al., 2001; Clemens and Hochman, 2004; Keeler et al., 2012; Johnson and Clemens, 2021).

### Dopaminergic mechanisms

#### Increased TH activity in the SN and striatum

Changes in TH activity are usually measured by immunohistochemistry or western blotting to determine the phosphorylated fraction of total TH in the corresponding brain areas (Connor et al., 2009). A transgenic mouse with tissue-specific overexpression of TH in catecholaminergic neurons has already been described and studied extensively (Kaneda et al., 1991; Kiuchi et al., 1993; Nakahara et al., 1993). However, unexpectedly, the significantly higher expression and activity of TH in this animal model were not accompanied by a significant increase in the basal striatal extracellular concentration of dopamine. This finding was attributed to compensatory adaptations. Therefore, to study the possible contribution of increased TH activity to the development of an RLS-like phenotype, it would be desirable to develop a conditional transgenic rodent that overexpresses TH during the postnatal or adult period in order to decrease the likelihood of compensatory developmental adaptations.

#### Decrease in striatal dopamine D2R binding potential

The density or expression of striatal D2R in rodents can be measured via several commonly used techniques, such as western blotting, immunohistochemistry, qPCR or *in situ* hybridization. The density and affinity of striatal D2R can be measured in radioligand-binding experiments. *In vivo* methods can also be used to measure striatal extracellular dopamine concentration, including microdialysis, cyclic voltammetry and fiber photometry. Although there are several pharmacological ways to increase striatal dopamine, one translationally sound mechanism would be to secondarily induce this by increasing dopamine synthesis (see above).

#### Increased BH4 and 3-OMD in the CSF

BH4 and 3-OMD are markers of increased L-DOPA synthesis in the brain, and can directly be analyzed in rodents. An increase in these markers might be secondary to an increase in TH activity, since 3-OMD is a byproduct of L-DOPA and BH4 is a TH co-factor. However, whether an increase in 3-OMD or BH4 in the brain is directly involved in RLS symptomatology has yet to be studied. An increase in brain BH4 can be produced by administering its natural precursor sepiapterin, as recently reported in humans (Smith et al., 2019).

### Exposure to CNS-active medications

#### Exposure to prescription SSRIs, antipsychotics or antihistamines

As already discussed in our previous guidelines (Salminen et al., 2021), SSRIs or other drugs can be applied by oral, systemic, intracranial or intrathecal administration, either acutely – to probe for possible fast effects – or continuously – by using slow-release miniature osmotic pumps. Intrathecal administration would distinguish between spinal and supra-spinal mechanisms.

#### Exposure to L-DOPA

To reproduce augmentation in a rodent model of RLS, L-DOPA could be administered repeatedly or continuously via mini pumps, systemically or centrally, for prolonged periods that have yet to be established.

### Hypoxia and renal failure

#### Hypoxia

Hypoxia in rodents can be achieved by using different gas mixtures in individually ventilated cages. This can be done in the animal's home cage or in a separate chamber by ventilating the cage with a hypoxic gas mixture of ≤15% oxygen (O_2_) partial pressure. Mild hypoxia has previously been tested in a putative RLS animal model and did not elicit RLS-like signs in mice, either alone or in combination with non-anemic PID (Lo Martire et al., 2018).

#### Renal failure

Renal failure can be modeled in rodents by using three different strategies, the first of which is to a use a pre-existing genetic model of renal failure. Some of these models were developed to study primary podocyte-specific genetic focal segmental glomerulosclerosis (Matsusaka et al., 2005), HIV-associated nephropathy (Dickie et al., 1991) and Alport syndrome (Hanifa et al., 2019). The second strategy involves surgically induced renal failure by using sub-total nephrectomy (Hanifa et al., 2019), radiation necropathy (Robbins et al., 1995) or unilateral ureteral obstruction (Chevalier et al., 2009). The third strategy is to use spontaneous models of renal failure, which include the Buffalo/Mna rat (Nakamura et al., 1986), the Munich Wistar Frömter (MWF/Fub) rat (Fassi et al., 1998) and spontaneously hypertensive rat strains, such as the Wistar-derived one (Zhou and Frohlich, 2007).

### Other existing RLS rodent models

In addition to the constructs discussed above, several other approaches have been used to model RLS in rodents. We briefly mention these below and highlight why they have not been selected as having sufficient construct validity by this task force.

#### The A11 dopaminergic cell cluster lesion and other lesion models

The main mesencephalic ascending dopaminergic cell systems originate in the SN (pars compacta), the ventral tegmental area and the retrorubral field, which are located dorsally and caudally to the SN, and correspond to the originally described A9, A10 and A8 dopaminergic cell clusters. Additionally, there are other diencephalic dopaminergic cell clusters. In particular, the A11 hypothalamic cluster is the origin of descending spinal dopaminergic innervation (Clemens et al., 2006; Qu et al., 2006). The A11 model of RLS is based on the hypothesis that decreased dopamine function in the spinal cord mimics RLS-like phenotypes (Ondo et al., 2000; Clemens et al., 2006; Zhao et al., 2007; Lopes et al., 2012). However, an autopsy study of the brains of RLS patients found no evidence of degeneration of or biochemical changes, such as TH levels, to the A11 area of their brains (Earley et al., 2009). Nevertheless, a reduction in spinal monoaminergic innervation with age has been reported in rats (Ranson et al., 2003) and might contribute to the age-related increased prevalence of PLM. Furthermore, a significant role for spinal cord dopamine in RLS is supported by clinical evidence of therapeutic improvement in PLM, in patients with spinal cord injury treated with a dopamine agonist (Earley et al., 2001; Kumru et al., 2015). In addition to the A11 hypothalamic cluster, targeted lesions of the corticospinal and rubrospinal tracts and their afferent sources have also been reported to induce RLS-like movements (Guo et al., 2017).

#### Opioid receptor knockout models

Genetic inactivation of the µ-opioid receptors in mice has recently been shown to reproduce some RLS-like behavioral phenotypes (Lyu et al., 2019b). However, despite the therapeutic effects of opioids, only one pilot study has shown some alterations in the levels of enkephalins and dynorphins in the thalamus and SN regions of RLS patient brains (Walters et al., 2009). Furthermore, opioid receptors are as yet not genetically associated with RLS. Therefore, the task force concluded that the validity of opioid system modifications as a construct for RLS was not sufficiently supported by the currently available evidence.

## Conclusions

These guidelines for the construct validity of RLS animal models can be used to evaluate previously published research and to generate novel RLS rodent models. The translation of the discussed RLS risk and triggering factors and pathophysiological mechanisms to rodent models requires researchers to identify the most appropriate methodologies to induce or replicate these factors in animals. Genetically modified rodents have already been studied individually for their ability to recapitulate several RLS pathogenetic mechanisms and RLS-like behavioral phenotypes (DeAndrade et al., 2012; Drgonova et al., 2015; Salminen et al., 2017, 2019; Lyu et al., 2019a, 2020a). In addition, PID during the post-weaning period – either alone or in combination with hypoxia or pregnancy – has been used to reproduce the RLS-like behavioral phenotype in rodents (Mariano et al., 2014; Lo Martire et al., 2018; Lai et al., 2017). Other potential methods, such as renal failure and CNS-active medications, remain to be studied in the context of RLS. In the future, these factors should be studied in compound study designs, where, for example, a genetic model is exposed to PID or renal failure. Based on the cumulative effects of different risk factors in humans (Trenkwalder et al., 2016), this would be expected to strengthen the construct validity of the rodent model of RLS and may ultimately lead to a better interpretation and a more widely accepted recognition of the mechanisms underlying RLS.

The analysis and study of the different putative pathophysiological mechanisms in isolation are also encouraged. This approach could causally link these mechanisms to a specific RLS-like behavioral phenotype and investigate their impact and role in the chain of pathophysiological events. The appearance of a specific pathophysiological mechanism upon the manipulation or induction of an RLS risk or triggering factor or pathophysiological mechanism would strengthen our understanding of the disease's progression. Successful examples of this approach include the use of dietary-induced BID in rodents, which recapitulates several dopaminergic and electrophysiological RLS features (Earley et al., 2014; Connor et al., 2009; Ferré et al., 2021) and the global or conditional cortical KO of the *Btbd9* gene, whose protein product enhances motor cortico-striatal transmission in mice (Lyu et al., 2020a).

Rodent models established by using the constructs discussed here should also be investigated for their face validity following our guidelines for RLS-like behavior. Investigating RLS-linked factors separately and in different combinations will help to elucidate which specific risks, triggering factors and pathophysiological mechanisms lead to which RLS-like behavioral phenotypes. These guidelines do not promote the exclusive use of rodents, since other animal models, such as invertebrates (Box 2, Invertebrate animal models of RLS) and zebrafish (Spieler et al., 2014), and *in vitro* models (Gulyani et al., 2009) are also contributing to our understanding of RLS; these are, however, beyond the purview of the task force. Finally, these guidelines are based on the present understanding of RLS pathophysiology and should be revised in the future, as and when new data and approaches become available.

**Box 2. Invertebrate animal models of RLS**

Several single-nucleotide polymorphisms (SNPs) associated with restless leg syndrome (RLS) have been investigated in *Caenorhabditis elegans* and *Drosophila melanogaster*. While these organisms cannot recapitulate the complexity of human RLS, they might provide important clues about the role of genetic risk factors in the clinical phenotype of RLS. *C. elegans* has several orthologs of known RLS genetic risk factors, including *MEIS1*, *BTBD9* and *PTPRD* (*unc-62*, *hpo-9* and *ptp-3*, respectively) (Chen et al., 2019). It has eight identifiable dopaminergic neurons as well as orthologs of all the genes involved in dopamine synthesis, packaging, reuptake and metabolization (Jayanthi et al., 1998; Hobert, 2013). It also expresses several behavioral paradigms to evaluate dopaminergic function (Chen et al., 2019). The *dop-1* and *dop-3* genes encode for *C. elegans* dopamine receptors. HPO-9 and DOP-1 proteins function similarly in egg-laying and locomotor behaviors, and HPO-9 deficiency leads to increased transcription of *dop-3* (Lyu et al., 2020a). Both *unc-62* and *hpo-9* (Chen et al., 2022) are expressed in dopaminergic neurons, and suppression of *unc-62* impairs dopaminergic neuron terminal differentiation and dopamine pathway gene expression (Lyu et al., 2020b; Jimeno-Martín et al., 2022). Furthermore, interference with *unc-62* expression increases expression of ferritin, indicating a possible connection between the *MEIS1* gene and iron metabolism in humans (Catoire et al., 2011).

*D. melanogaster* has a highly regulated rest/activity cycle of ∼24 h and has been extensively used to investigate sleep disorders (Donelson and Sanyal, 2015). It has a single *BTBD9* ortholog (locus tag CG1826), the genetic deletion of which mildly shortens lifespan and produces RLS-like phenotypes, including increased locomotor activity, significant sleep fragmentation and responsiveness to dopamine agonists, such as pramipexole (Freeman et al., 2012).
